# In vivo evidence of remote neural degeneration in the lumbar enlargement after cervical injury

**DOI:** 10.1212/WNL.0000000000007137

**Published:** 2019-03-19

**Authors:** Gergely David, Maryam Seif, Eveline Huber, Markus Hupp, Jan Rosner, Volker Dietz, Nikolaus Weiskopf, Siawoosh Mohammadi, Patrick Freund

**Affiliations:** From the Spinal Cord Injury Center Balgrist (G.D., M.S., E.H., M.H., J.R., V.D., P.F.), University Hospital Zurich, University of Zurich, Switzerland; Wellcome Trust Centre for Neuroimaging (N.W., S.M., P.F.), UCL Institute of Neurology, London, UK; Department of Neurophysics (M.S., N.W., P.F.), Max Planck Institute for Human Cognitive and Brain Sciences, Leipzig; and Department of Systems Neuroscience (S.M.), University Medical Center Hamburg-Eppendorf, Hamburg, Germany.

## Abstract

**Objective:**

To characterize remote secondary neurodegeneration of spinal tracts and neurons below a cervical spinal cord injury (SCI) and its relation to the severity of injury, the integrity of efferent and afferent pathways, and clinical impairment.

**Methods:**

A comprehensive high-resolution MRI protocol was acquired in 17 traumatic cervical SCI patients and 14 controls at 3T. At the cervical lesion, a sagittal T2-weighted scan provided information on the width of preserved midsagittal tissue bridges. In the lumbar enlargement, high-resolution T2*-weighted and diffusion-weighted scans were used to calculate tissue-specific cross-sectional areas and diffusion indices, respectively. Regression analyses determined associations between MRI readouts and the electrophysiologic and clinical measures.

**Results:**

At the cervical injury level, preserved midsagittal tissue bridges were present in the majority of patients. In the lumbar enlargement, neurodegeneration—in terms of macrostructural and microstructural MRI changes—was evident in the white matter and ventral and dorsal horns. Patients with thinner midsagittal tissue bridges had smaller ventral horn area, higher radial diffusivity in the gray matter, smaller motor evoked potential amplitude from the lower extremities, and lower motor score. In addition, smaller width of midsagittal tissue bridges was also associated with smaller tibialis sensory evoked potential amplitude and lower light-touch score.

**Conclusions:**

This study shows extensive tissue-specific cord pathology in infralesional spinal networks following cervical SCI, its magnitude relating to lesion severity, electrophysiologic integrity, and clinical impairment of the lower extremity. The clinical eloquence of remote neurodegenerative changes speaks to the application of neuroimaging biomarkers in diagnostic workup and planning of clinical trials.

Besides the primary damage to the lesion site, traumatic spinal cord injury (SCI) triggers a cascade of pathologic processes remote from injury.^1,2^ These are reflected by neuronal dysfunction, such as premature exhaustion of motor neurons and impaired sensorimotor function below the level of the lesion.^3–9^ Moreover, remote dynamic neurodegenerative and reorganizational processes of the neural circuits are believed to play a critical role in the patients' long-term recovery^1^ and might determine the success of future regenerative therapies.

To better understand the interaction between degenerative processes at and caudal to a cervical lesion and their relation to electrophysiologic and clinical measures of the lower extremity, 3 questions were investigated: (1) Are the degenerative processes in the lumbar enlargement similar to those demonstrated within the high cervical cord above the injury?^10^ (2) Is there a relationship between lesion severity and the magnitude of neurodegeneration in the lumbar enlargement? (3) Is there a relationship between structural changes at and below the level of injury and electrophysiologic and clinical measures?

We applied high-resolution multimodal MRI to the lumbar enlargement^11,12^ and quantified electrophysiologic and clinical measures of lower limb to investigate tissue-specific cord pathology in chronic cervical SCI patients. We hypothesized that (1) remote tissue-specific neurodegeneration (reflected by macrostructural and microstructural MRI changes) occurs in the lumbar enlargement, (2) preserved midsagittal tissue bridges at the lesion site are related to the magnitude of tissue-specific cord pathology in the lumbar enlargement, and (3) preserved midsagittal tissue bridges and remote tissue-specific neurodegeneration correlate with electrophysiologic and clinical measures of lower limb function.

## Methods

### Standard protocol approvals, registrations, and patient consents

The study protocol was designed in accordance with the Declaration of Helsinki and was approved by the local ethics committee (EK-2010-0271). All participants provided written informed consent prior to study enrollment.

### Participants

Seventeen SCI patients (4 female, age 42.7 ± 14.0 years [mean ± SD]) were recruited and admitted to the outpatient clinic at Balgrist University Hospital, Zurich, Switzerland, between August 19, 2015, and December 6, 2016. In addition, 14 healthy volunteers (4 female, age 42.4 ± 17.2 years) formed the control dataset. SCI patients fulfilled the following inclusion criteria: (1) traumatic cervical SCI, (2) no other neurologic or psychiatric disorders, (3) no MRI contraindications, and (4) no pregnancy.

### Clinical examination

In patients, neurologic impairment was assessed by means of the International Standards for Neurologic Classification of Spinal Cord Injury (ISNCSCI) protocol.^13^ Single motor and sensory scores in the ISNCSCI scoring sheet were summed up between L2 and S4-5 neurologic levels and are referred to as lower extremity motor (LEMS), light touch (LELT), and pinprick scores (LEPP) throughout the article. Daily life independence (i.e., self-care, respiration, sphincter management, and mobility) was assessed by the Spinal Cord Independence Measure (SCIM).^14^

### Electrophysiologic measurements

The electrophysiologic examinations were conducted according to the standard protocol of the European Multicenter Study about Spinal Cord Injury (emsci.org/). Abductor hallucis (AH) and tibialis anterior (TA) motor evoked potentials (MEP) were acquired simultaneously by single-pulse transcranial magnetic stimulation, placing the coil at 4 cm rostral of Cz, provoking a response in the AH and TA muscles. A sample frequency of 2,000 Hz, biphasic stimulus duration of 200 μs, and a band-pass filter of 30 Hz–1 kHz were used. The time from the stimulation to the muscle response onset determined the MEP latency and the amplitude was measured from baseline to the highest negative peak of the potential.

To obtain tibial sensory evoked potential (SEP), posterior tibial nerves were stimulated bilaterally at the ankle. The stimulation was performed until a motor response was induced. Cortical responses were recorded with active electrode at Cz' (2 cm posterior to Cz) and referenced to Fz according to the 10–20 EEG system. The impedance was maintained under 5 kΩ. Two sets of 150 responses were averaged and superimposed. The SEP P40 latency was measured as the time from the stimulation to the first positive peak of the primary complex, and the amplitude as the difference between the P40 and N50 (first negative) peaks.

Means of both sides (left and right) of the electrophysiologic measures were used for analysis, as MRI readouts were extracted from the whole cross-section of the spinal cord (SC). Evoked potential latencies were normalized for height. For MEP and SEP, patients without any recordable potential were given an amplitude of 0 mV/μV. Only patients with bilateral responses were included into the latency analysis. Due to the small number of participants with bilateral MEP responses, MEP latency analysis was not performed.

### Image acquisition

All MRI measurements were performed on a clinical 3T Siemens (Erlangen, Germany) Skyra^Fit^ system, using a standard radiofrequency body coil for transmission and the combination of 16-channel radiofrequency head and neck coil and standard spine matrix coil for reception. Foam positioners were placed under the knees to reduce the normal cord lordosis and maximize the contact between the spine coil and the lower SC. In addition, an MRI-compatible cervical collar (Laerdal Medical, Stavanger, Norway) was used to reduce motion in the cervical cord.^15^

At the lesion level, a standard clinical sagittal T2-weighted image was acquired to assess the extent of the lesion. The following settings were used: repetition time (TR) 3,430 ms, echo time (TE) 90 ms, flip angle 150°, field of view (FOV) 218 × 218 × 55 mm^3^, resolution 0.34 × 0.34 × 2.75 mm^3^.

In the lumbar enlargement, high-resolution structural data were acquired using a T2*-weighed 3D multi-echo gradient-echo sequence (multi-echo data image reconstruction sequence). The 20 axial–oblique slices were centered at the widest point of the enlargement as appearing in a localizing sagittal T2-weighted image, following the procedure described in Yiannakas et al.^11^ Depending on the participant, the widest point was located between the T10 and L1 vertebral levels. Four measurements were acquired using the following parameters: slice thickness 2.5 mm, in-plane resolution 0.5 × 0.5 mm^3^, in-plane FOV 192 × 162 mm^2^, TE 19 ms, TR 44 ms, flip angle 11°, readout bandwidth 260 Hz/pixel, 4 measurements, total acquisition time 8:32 minutes. Zero filling interpolation was used to double the apparent in-plane resolution (0.25 × 0.25 mm^2^).

A diffusion MRI dataset consisting of 60 diffusion-weighted (b = 500 seconds/mm^2^) and 7 T2-weighted (b = 0 seconds/mm^2^) volumes was also acquired using a reduced-FOV single-shot spin-echo echo-planar imaging sequence with identical slice prescription as the T2*-weighed images. Acquisition measures were as follows: slice thickness 5 mm (10% gap), in-plane resolution 0.76 × 0.76 mm^2^, in-plane FOV 133 × 30 mm^2^, TE 71 ms, TR 350 ms, 5/8 partial Fourier in phase-encoding direction (anterior–posterior direction). The acquisition time varied with the participant's heart rate with a nominal acquisition time of 5.2 minutes. The acquisition was cardiac gated (minimal duration between 2 successive triggers: 1,800 ms) to reduce artifacts related to CSF pulsation. The in-plane apparent resolution was doubled by zero filling interpolation (0.38 × 0.38 mm^2^).

### Image analysis

#### Lesion segmentation

In SCI patients, the lesion appearing as a hyperintense area in the T2-weighted image was segmented as previously described.^16^ On the midsagittal slice, we quantified the width of midsagittal tissue bridges,^16^ which was defined as the width of the normal-appearing tissue bundles ventral and dorsal to the visible lesion. The lesion characteristics could not be assessed in 4 out of 17 patients due to artifacts caused by the orthopedic fixations.

#### Processing of structural MRI data

An average of the 4 T2*-weighted volumes was created using serial longitudinal registration (SPM12, MATLAB 2013) to account for between-scan motion. The averaged image was resliced to 5 mm slice thickness to increase signal to noise ratio. Cross-sectional SC area (SCA) was obtained in all slices using the semi-automatic 3D active surface cord segmentation method as implemented in JIM 7.0.^17^ Three slices around the slice having the largest SCA were considered for further segmentation and analysis to ensure comparable and reproducible anatomical coverage of the lumbar enlargement.^11^

Gray matter (GM) was segmented manually (using sub-voxel segmentation in JIM 7.0) to determine cross-sectional GM area (GMA) measures. White matter area (WMA) was calculated as the difference between SCA and GMA. Using the same manual segmentation tools, GMA was further subdivided into bilateral dorsal (dGMA) and ventral horn (vGMA) areas as previously described.^10,18^ For the statistical analysis, the slice-averaged tissue areas including SCA, WMA, GMA, dGMA, and vGMA were used.

#### Processing of diffusion MRI data

We used the SPM-based ACID toolbox for processing the diffusion tensor imaging (DTI) data, following the procedure described in our previous studies.^19,20^ In short, we performed slice-wise registration between all 67 volumes to correct for motion and eddy-current artifacts. Then, we fitted the diffusion tensor model using a robust tensor fitting algorithm, which has been shown to effectively reduce physiologic artifacts, residual motion artifacts, and misregistrations.^20^ Tensor fitting generated the following DTI index maps: fractional anisotropy (FA), mean diffusivity (MD), axial diffusivity (AD), and radial diffusivity (RD). After tensor fitting, all DTI maps were registered to the corresponding T2*-weighed image using a nonlinear transformation (BSplineSyn algorithm^21^) implemented in the Spinal Cord Toolbox.^22^ The SC, GM, and white matter (WM) masks generated on the T2*-weighted structural image were then applied to the DTI maps and were manually adjusted if necessary to account for slight registration errors. All binary masks were one-voxel eroded to reduce partial volume effects. Finally, mean FA, MD, AD, and RD values were extracted from the SC, GM, and WM binary masks, which were used for subsequent analyses.

### Statistical analysis

All statistical analyses were performed in Stata 14 (StataCorp LP, TX). Age and sex differences between SCI patients and controls were assessed using Mann-Whitney *U* test and Fisher exact test, respectively. We excluded 4 SCI patients and 1 healthy control from the cross-sectional area and DTI measurements due to extensive motion artifacts or signal dropout. First, to assess remote tissue-specific neurodegeneration, cross-sectional tissue areas (SCA, WMA, GMA, vGMA, and dGMA) and DTI measures (FA, MD, AD, RD) were compared between SCI patients and controls using a 2-sample *t* test (1-tailed, unequal variances, α = 0.05). Second, correlation analysis was used to investigate linear associations between these remote MRI readouts and lesion measures (α = 0.05). Finally, the relationships between the MRI readouts (lesion-level and in the lumbar enlargement), electrophysiologic assessments, and clinical scores were assessed using correlation analysis (α = 0.05).

### Data availability

The authors certify they have documented all data, methods, and materials used to conduct the research presented. Anonymized data pertaining to the research presented will be made available by request from qualified investigators.

## Results

### Demographic, clinical, electrophysiologic, and radiologic characteristics

There was no significant difference between the SCI patients and controls in terms of sex (Fisher exact test, *p* = 0.370) and age (Mann-Whitney *U* test, z = −0.437, *p* = 0.66). Of the 17 patients, 3 were classified as American Spinal Injury Association Impairment Scale (AIS) A, 4 as AIS C, and 10 as AIS D (table 1). Patients were scanned on average 74.5 ± 60.0 months following the injury. Midsagittal tissue bridges were present in all incomplete patients (AIS B-D) and in 2/3 complete patients (AIS A). The amplitudes of the recorded signals are shown in table 1. No bilateral AH and TA MEP signal was detected in motor complete patients, while 2 out of 8 motor incomplete patients did not have bilateral TA MEP signal. Tibial SEP (tSEP) signal was not recordable in complete and 3 out of 12 incomplete patients. The mean (±SD) amplitudes for AH and TA MEP, as well as tSEP, were 0.43 ± 0.48 mV, 0.15 ± 0.19 mV, and 0.85 ± 1.11 μV, respectively. The mean latencies for AH MEP, TA MEP, and tSEP were 44.41 ± 2.71, 34.05 ± 1.88, and 44.13 ± 7.18 ms, respectively.

**Table 1:**
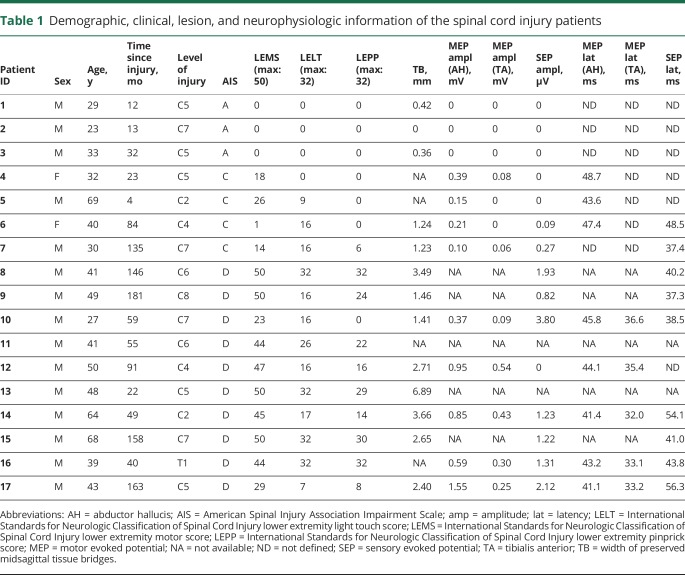
Demographic, clinical, lesion, and neurophysiologic information of the spinal cord injury patients

### Remote tissue-specific neurodegeneration in the lumbar enlargement

**Figure 1:**
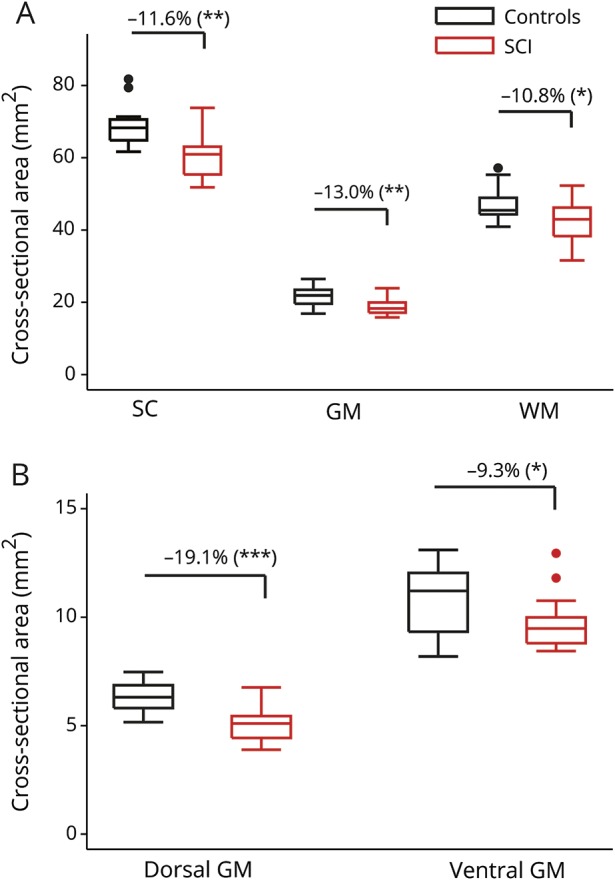
Box plots of tissue-specific cross-sectional areas in the lumbar enlargement Spinal cord (SC), gray matter (GM), and white matter (WM) areas are illustrated in (A), while dorsal GM and ventral GM areas resulting from GM subsegmentation are plotted in (B). In case of significant group-level difference (*p* < 0.05), the percent group difference is also indicated. Both WM and GM were significantly smaller in patients (−10.8% and −13.0%, respectively), where dorsal GM contributed proportionally more to the GM atrophy. Dots represent outliers that fall below Q1 − 1.5 × interquartile range (IQR) or above Q3 + 1.5 × IQR (Q1, Q3 = first and third quartiles, respectively; IQR = Q3 − Q1).

**Figure 2:**
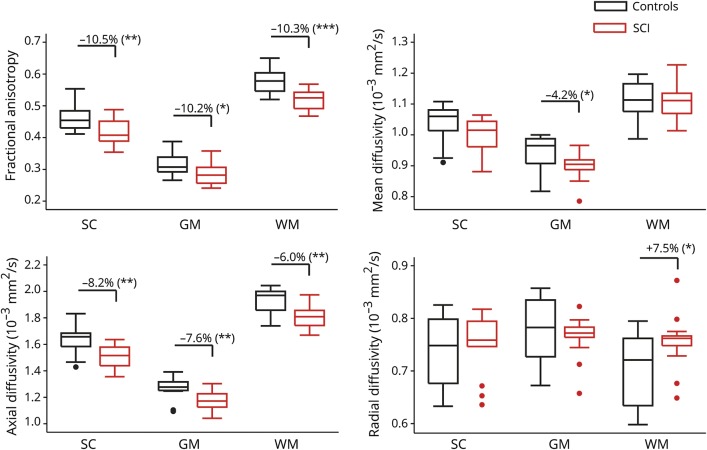
Box plots of tissue-specific diffusion tensor imaging scalar values (fractional anisotropy [FA], mean diffusivity [MD], axial diffusivity [AD], radial diffusivity [RD]) comparing controls and patients In case of significant group-level difference (*p* < 0.05), the percent group difference is also reported. Spinal cord injury (SCI) patients had lower FA and AD and higher RD in the white matter (WM). In the gray matter (GM), patients had lower FA, MD, and AD. Dots represent outliers that fall below Q1 − 1.5 × interquartile range (IQR) or above Q3 + 1.5 × IQR (Q1, Q3 = first and third quartiles, respectively; IQR = Q3 − Q1).

In the lumbar enlargement, SCI patients had lower WMA (−10.8%, *p* = 0.011) and GMA (−13.0%, *p* = 0.005) compared to controls, with both ventral GMA (−9.3%, *p* = 0.046) and dorsal GMA (−19.1%, *p* < 0.001) being affected (figure 1 and table 2). In the atrophied lumbar enlargement, patients had lower FA and AD values in both GM and WM compared to controls (figure 2 and table 2). In addition, higher RD of WM and lower MD of GM were observed in SCI patients.

**Table 2:**
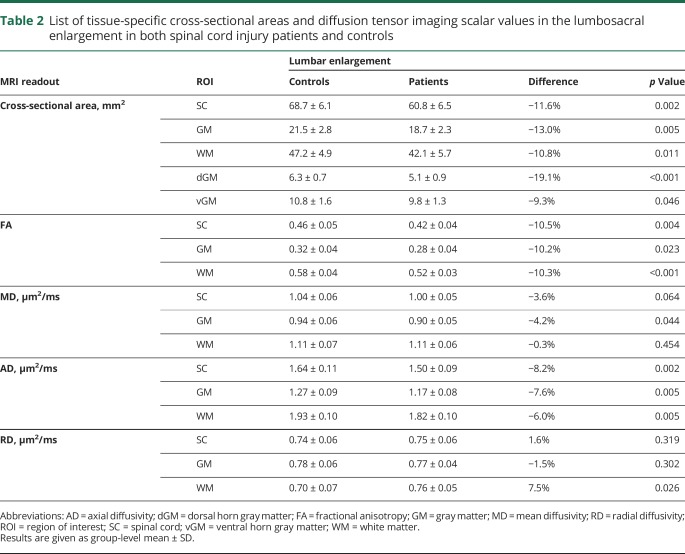
List of tissue-specific cross-sectional areas and diffusion tensor imaging scalar values in the lumbosacral enlargement in both spinal cord injury patients and controls

### Relation of tissue bridges to cord pathology

**Figure 3:**
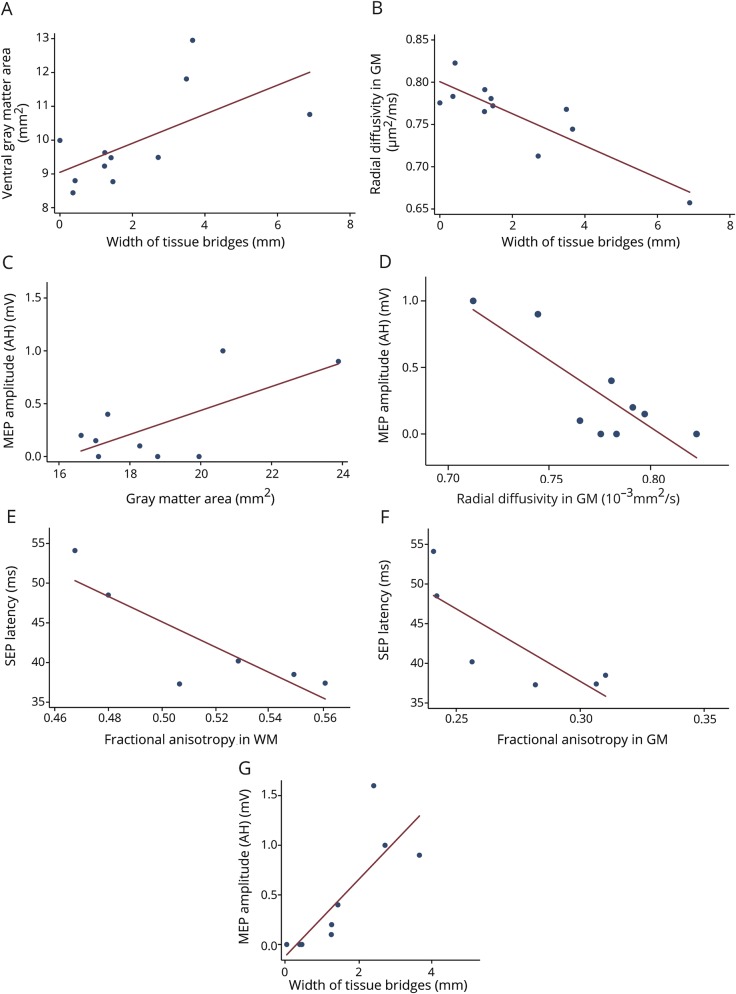
Significant associations between the severity of the lesion (width of midsaggital tissue bridges), remote structural changes in the lumbar enlargement, and electrophysiologic measures (A, B) Width of midsaggital tissue bridges is associated with cross-sectional gray matter (GM) area and radial diffusivity in the GM of the lumbosacral enlargement. (C, D) GM area and radial diffusivity of GM are associated with motor evoked potential (MEP) amplitudes. (E, F) Fractional anisotropy in the white matter (WM) and GM is associated with sensory evoked potential (SEP) latencies. (G) Width of tissue bridges is associated with the MEP amplitudes. AH = abductor hallucis.

Patients with larger width of the midsagittal tissue bridges had larger vGMA (*r* = 0.62, *p* = 0.041) (figure 3A) and lower MD (WM: *r* = −0.76, *p* = 0.006; GM: *r* = −0.86, *p* < 0.001) and RD (WM: *r* = −0.71, *p* = 0.015; GM: *r* = −0.87, *p* < 0.001) (figure 3B).

### Relationship between tissue bridges, cord pathology, and electrophysiologic measures

Patients with larger width of midsagittal tissue bridges had larger MEP amplitudes (AH: *r* = 0.81, *p* = 0.008; TA: *r* = 0.908, *p* < 0.001) (figure 3G), but not SEP amplitudes (*r* = 0.41, *p* = 0.192). In addition, smaller MEP amplitudes were associated with smaller GMA (AH: *r* = 0.68, *p* = 0.043; TA: *r* = 0.75, *p* = 0.020) and higher RD in the GM (AH: *r* = −0.83, *p* = 0.006; TA: *r* = −0.89, *p* = 0.001) (figure 3, C and D). Patients with longer SEP latencies had lower FA in both WM and GM (WM: *r* = −0.85, *p* = 0.030; GM: *r* = −0.81, *p* = 0.049) (figure 3, E and F).

### Relation of lesion measures and cord pathology to clinical outcome

**Figure 4:**
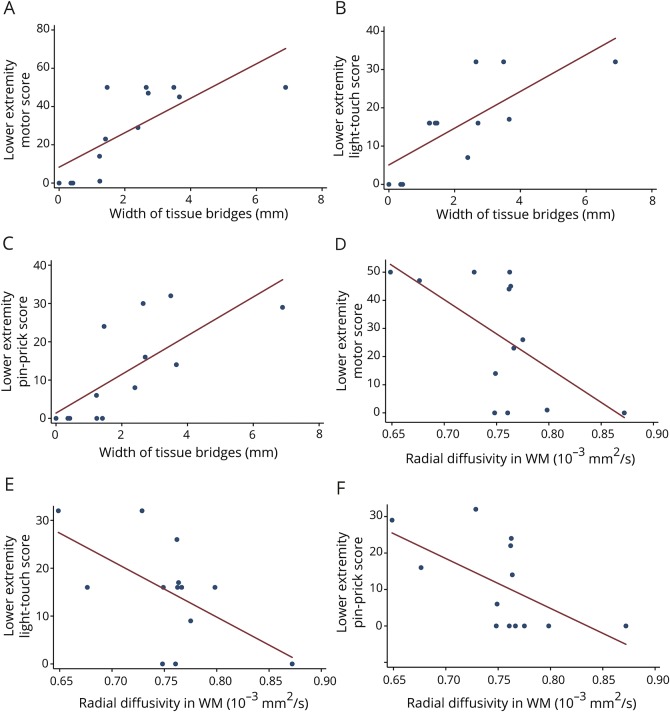
Significant associations between the severity of the lesion (width of midsaggital tissue bridges), remote structural changes in the lumbosacral enlargement, and clinical outcome (A–C) Width of midsaggital tissue bridges is associated with ISNCSCI extremity motor, light touch, and pinprick scores. (D–F) Radial diffusivity in the white matter (WM) correlates with lower extremity motor, light touch, and pinprick scores.

At the lesion level, the width of midsagittal tissue bridges correlated positively with LEMS (*r* = 0.75, *p* = 0.003), LELT (*r* = 0.76, *p* = 0.003), LEPP (*r* = 0.73, *p* = 0.046) (figure 4, A–C), and SCIM (*r* = 0.70, *p* = 0.017). In the lumbar enlargement, RD of both GM and WM were negatively correlated with LEMS (WM: *r* = −0.60, *p* = 0.029; GM: *r* = −0.63, *p* = 0.021), LELT (WM: *r* = −0.58, *p* = 0.039; GM: *r* = −0.62, *p* = 0.025), LEPP (WM: *r* = −0.59, *p* = 0.033; GM: *r* = −0.63, *p* = 0.020), and SCIM (WM: *r* = −0.61, *p* = 0.045; GM: *r* = −0.70, *p* = 0.017; figure 4, D–F). In addition, MD of the WM correlated negatively with LEMS (*r* = −0.72, *p* = 0.006), LELT (*r* = −0.61, *p* = 0.028), LEPP (*r* = −0.69, *p* = 0.009), and SCIM (*r* = −0.65, *p* = 0.032).

## Discussion

While the extent of secondary remote changes in the cervical cord after traumatic SCI has been described in vivo, this study shows in vivo evidence of secondary remote neurodegenerative changes affecting infralesional spinal networks. We observed marked macrostructural (reflected by cross-sectional area measurements) and microstructural (reflected by diffusion MRI) signs of degeneration in both the GM and WM within the lumbar enlargement. The magnitude of (transsynaptic) neurodegeneration was associated with changes in preserved electrophysiologic information flow of afferent and efferent pathways and lower limb function. Thus, next to tissue-specific supralesional cord pathology,^10,23,24^ the lumbar enlargement after a cervical SCI also undergoes neurodegenerative changes, both in WM and GM tissue.

### Remote tissue-specific neurodegeneration in the lumbar enlargement

**Figure 5:**
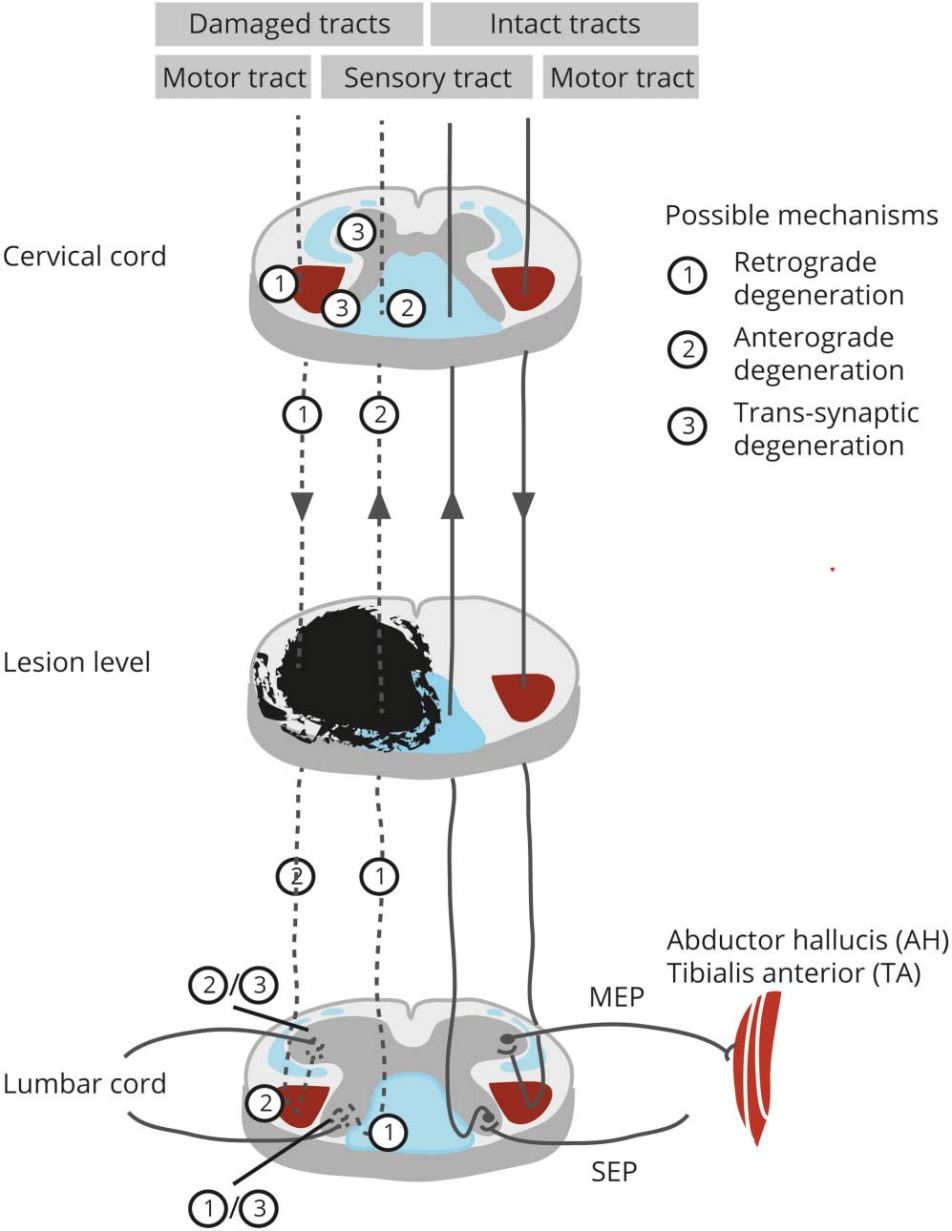
Secondary degenerative processes occurring remotely, above and below the primary injury site Sensory and motor tracts affected by the injury undergo anterograde or retrograde (depending on the direction) axonal degeneration and accompanying demyelination. In the lumbar cord, the lower motor neurons located in the ventral horn may undergo transsynaptic degeneration due to the loss of input from the injured corticospinal tracts. Similarly, second-order sensory neurons of the spinothalamic and dorsal column medial lemniscus systems can also be affected by transsynaptic degeneration. MEP = motor evoked potential; SEP = sensory evoked potential.

In the WM of the lumbar enlargement, AD was decreased and RD increased, which led to the overall reduction in FA. Furthermore, impaired microstructure also translated to tissue atrophy in the WM. These observations are consistent with previous studies that showed that axonal degeneration, including disintegration of the axonal skeleton and membrane, as well as accompanying demyelination leads to increased RD and decreased AD after experimental or human SCI.^25–27^ This suggests that the dominant histopathologic substrates of the observed in vivo human WM changes are likely to be anterograde and retrograde degeneration of descending motor pathways and ascending afferent spinal projections (for an overview of WM degeneration processes, see figure 5). Moreover, accumulation of cellular debris in the extracellular space could further reorganize the microstructural architecture and lead to reduced anisotropy and diffusivity in the WM.^28^ Interestingly, the magnitude of neurodegeneration in the lumbar enlargement was similar to the above-level neurodegeneration in the upper cervical cord.^10^

In the GM of the lumbar enlargement, we found decreased AD, which led to reduction in MD and FA. In the acute and subacute phases, animal SCI models have demonstrated morphologic changes in the GM including decreased number but increased length of the remaining dendrites and enlarged soma size.^29,30^ In humans, however, remote structural changes in the cords' GM after SCI are understudied. The atrophy of ventral horns presumably reflects transsynaptic degeneration of flexor motor neuron pool due to deprivation from supraspinal input (figure 5).^7,31,32^ In contrast, the extensor motor neuron pool in the lumbar cord continues to receive proprioceptive input to become activated. It has been reported that the extensor neurons are likely to survive after the injury compared to flexor neurons.^5^ Similarly, dorsal horn neurons, interneurons, and propriospinal networks^33^ are also prone to undergo a partially transsynaptic degeneration (figure 5). Other mechanisms including vascular remodeling and changes in the amount of glial cells^34^ could also play a role in GM pathology; however, their degree of contribution is not clear.

### Relation of tissue bridges to cord pathology

Following the initial injury, a post-traumatic cyst develops in the majority of patients within the first month.^16^ It has been shown that the magnitude of such neuronal tissue loss (i.e., lesion severity) is associated with reduced cross-sectional GM and WM area above the level of lesion.^10^ In this study, we show relationships between lesion severity (i.e., preserved midsaggital tissue bridges) and remote cord pathology below the level of injury. We found associations between the width of the midsagittal tissue bridges and the magnitude of ventral horn atrophy as well as microstructural alterations within the lumbar enlargement. Thus, the relation between the severity of lesion and below-level neurodegeneration indicates that the initial damage to the cervical cord primarily drives (initiates) remote neurodegenerative processes.^35,36^

### Relationship between tissue bridges, cord pathology, and electrophysiologic measures

Electrophysiologic measures obtained after SCI are predictive of functional recovery.^37^ We previously demonstrated that preserved tissue bridges underwrite electrophysiologic communication.^16^ This finding has been confirmed in our data: (1) no MEP and SEP signals were present in the patient without tissue bridges and (2) patients with larger width of tissue bridges also had larger MEP amplitudes. However, here we show that not only does the severity of lesion correlate with the cervical impairment of conductivity, but it is also associated with the tissue-specific pathology in the lumbar enlargement. That is, patients with GM atrophy and altered microstructure (reflected by RD and MD) within the GM had lower MEP amplitudes of the abductor halluces (extensor) and tibialis anterior (flexor) muscles. These associations are thought to reflect transsynaptic changes within both extensor and flexor motor neuron pools. Based on the literature, we suggest that the leg extensor motoneurons are less affected as they continue to receive proprioceptive input, even after the injury, while a loss of flexor motoneurons occurs after deprivation from supraspinal input.^5,31^ Thus, a great part of the neurodegenerative changes observed within the GM might directly relate to the loss of supraspinal drive onto the flexor motor neuron pools.

### Relation of lesion measures and cord pathology to clinical outcome

The width of midsagittal tissue bridges measured at 1 month post-SCI predicts clinical recovery at 1 year post-SCI.^16^ In addition, chronic SCI patients show an association between cervical macrostructural and microstructural changes and clinical impairment.^10^ Our findings are in agreement with this observation: patients with smaller width of midsagittal tissue bridges had greater functional impairment below the lesion. While several DTI measures in the WM (MD, RD) and GM (MD, RD) were related significantly to the motor impairment, no correlation was found between the ventral horn area in the lumbar enlargement and the ISNCSCI motor score. The reason for this might be that motor scores merely reflect muscle strength quantitatively on a coarse 5-grade scale. Hence, macrostructural changes within the ventral horn can hardly be translated into measurable functional loss.

### Limitations

The study had several limitations. First, this cross-sectional study included a heterogeneous patient cohort in terms of injury severity, time since injury, and lesion level. In addition, MRI readouts in the spinal cord have been shown to alter with age and sex,^38^ which might have affected the groupwise comparisons between DTI measures. To address this, we recruited sex- and age-matched controls. Furthermore, the expected sex- and age-related effects were smaller than the SCI-induced ones.

Another limitation is related to the technical feasibility of lumbar cord imaging such as the low signal-to-noise ratio (SNR), susceptibility, motion, and other physiologic artifacts. We have addressed these issues at the acquisition and image processing stage. To compensate for the relatively low SNR of the lumbar images due to the application of a spine matrix coil (instead of head and neck surface coil in the cervical coil), we have (1) placed foam positioners under the knee to increase the contact between the spine and the coil, (2) acquired 4 averages for the T2*-weighted and relatively many volumes (67) for the DTI dataset, and (3) acquired thick slices (5 mm). To compensate for the susceptibility artifacts affecting mainly the DTI dataset, we have coregistered the distorted DTI dataset to the nondistorted T2*-weighted images. Motion artifacts were probably the biggest issue, which resulted in the exclusion of 4 patients and 1 healthy control due to extensive blurring. In addition, we used cardiac gating for the DTI dataset to minimize the effect of CSF pulsation. In all participants, between-volume motion was corrected by realigning the T2*-weighted and DTI images using serial longitudinal registration and slice-wise linear registration, respectively. Finally, the application of robust tensor fitting further reduced residual motion artifacts in the DTI dataset, as previously demonstrated.^20^

At present, automatic segmentation algorithms have been validated only for the cervical GM.^39^ Although the segmentation of the cross-sectional area of the lumbar cord was semi-automatic (only the midpoint of the cord had to be set manually), the GM was segmented manually. To be as accurate and reliable as possible in the region of interest (ROI) analysis, (1) GM was segmented on the T2*-weighted image due to the better contrast and applied on the DTI dataset after coregistration, (2) all the segmentation was performed by the same experienced user, (3) all SC, GM, and WM masks were 1-voxel eroded to reduce partial volume effects, and (4) all final masks were visually inspected and corrected if necessary. However, despite the careful approach applied in the ROI selection, remaining partial volume effects and imperfect registration could have affected the ROI-based analysis, but we considered their extent to be minor.

## Discussion

Tracking trauma-induced tissue-specific neurodegenerative and reorganizational changes in infralesional spinal networks demonstrates the far-reaching consequences of a focal CNS injury. Our findings suggest that the macrostructural and microstructural changes reflect signs of transsynaptic degeneration in sensorimotor pathways. These are for example reflected in the premature exhaustion of motoneurons and an impaired sensorimotor function below the lesion level.^4,5,7^ The clinical consequence of remote neurodegenerative changes—including axonal and transsynaptic changes—favors the application of multimodal MRI approaches in routine clinical decision-making and planning of clinical trials. Neuroimaging biomarkers of remote cord pathology offer efficient targeting of therapeutic agents and monitoring in clinical trials.

## Glossary

AD: axial diffusivity
AH: abductor hallucis
AIS: American Spinal Injury Association Impairment Scale
dGMA: dorsal gray matter area
DTI: diffusion tensor imaging
FA: fractional anisotropy
FOV: field of view
GM: gray matter
GMA: gray matter area
ISNCSCI: International Standards for Neurologic Classification of Spinal Cord Injury
LELT: International Standards for Neurologic Classification of Spinal Cord Injury lower extremity light touch score
LEMS: International Standards for Neurologic Classification of Spinal Cord Injury lower extremity motor score
LEPP: International Standards for Neurologic Classification of Spinal Cord Injury lower extremity pinprick score
MD: mean diffusivity
MEP: motor evoked potential
RD: radial diffusivity
ROI: region of interest
SC: spinal cord
SCA: spinal cord area
SCI: spinal cord injury
SCIM: Spinal Cord Independence Measure
SEP: sensory evoked potential
SNR: signal-to-noise ratio
TA: tibialis anterior
TE: echo time
TR: repetition time
tSEP: tibial sensory evoked potential
vGMA: ventral horn gray matter area
WM: white matter
WMA: white matter area
